# A chitosan fiber as green material for removing Cr(VI) ions and Cu(II) ions pollutants

**DOI:** 10.1038/s41598-021-02399-5

**Published:** 2021-11-25

**Authors:** Shujie Zhang, Yating Zhang, Lisong Fu, Mengke Jing

**Affiliations:** School of Textile Science and Engineering, Tiangong University, Tianjin, 300387 China

**Keywords:** Environmental chemistry, Environmental impact

## Abstract

The application shell uses cellulose as a green and recyclable fiber material, which has great value in the field of water treatment environment. Varying factors, including pH value, dosage of CS, reaction time and original Cr(VI) ions and Cu(II) ions were studied to investigate the Cr(VI) and Cu(II) ions removal efficiency. The obtained shell trichlorocellulose has better permeability to copper ions, which is mainly due to the different oxide states of copper ions and chromium ions in a pH environment, which lead to different combinations. The price of shell cellulose neutralization is relatively low. Metal ions have better absorption properties. The kinetic and thermodynamic characteristics of the adsorption process of copper ions by chitosan yarns were discussed. The adsorption process of copper ions conformed to the quasi-second-order kinetic equation. It can be fitted by Langmuir isotherm. The adsorption of copper ions by the yarn is a spontaneous thermal reaction with both physical adsorption and chemical adsorption. Compared with chromium ions, chitosan fibers have better adsorption of copper ions, which is mainly because the amino groups in chitosan fibers can have good chelation with copper ions. SEM, FTIR, XRD were used to characterize the adsorption of copper ions by chitosan fibers, and the mechanism of the adsorption of metal ions by chitosan fibers was explored.

## Introduction

With the rapid development of industrial economy, heavy metals such as copper and chromium contained in industrial wastewater will cause serious environmental pollution^1–5^. Heavy metals can cause serious harm to the human body, such as chromium: it can cause numbness of the limbs and mental disorders, and copper can cause arthritis and accelerate human aging^6–10^. The concept of "cleaner production" was put forward in the 1950s and 1960s. The concept was to quickly realize the impact of industrial development on the environment, thereby establishing a safe and friendly environmental concept. Now that the concept of environmental protection has been deeply rooted in the hearts of the people, economic development is gradually pursuing green^11–17^.

At present, many treatment methods are applied to remove heavy metals, includingg membrane separation, chemical precipitation, solvent extraction, ion-exchange, reduction, reverse osmosis and adsorption^1,18^, The adsorption method is widely used for its good results with the advantages of non-production of potential secondary pollutants, recyclable usage, easy accessibility and high efficient usage. Chitosan is widely used in the fields of food, medicine and wastewater due to its excellent biocompatibility, biodegradability, non-toxicity, adsorption and antibacterial properties. The chitosan composite prepared by Zhuang^19^ and others by the wet spinning method has good adsorption of cobalt ions. Fenglei Liu^20^ et al. prepared viscose fibers functionalized with chitosan that can adsorb gold well. Ruihua Mu et al.^21^ prepared a porous lignosulfonate/chitosan adsorbent, which has good adsorption properties for copper ions and cobalt ions.

Chitosan fiber is a kind of green material, dissolved in acidic spinning solution, has good antibacterial properties and adsorption to metal ions, and can be recycled. In the previous research on the removal of heavy metal ions in wastewater, chitosan and modified substances were mostly used as adsorbents, and the research and application of chitosan fibers were very few. In this paper, chitosan fiber is used as the experimental material to discuss the influence of chitosan fiber, metal chromium ions and copper ions on reaction time, copper ion concentration, temperature and pH value. The kinetic and thermodynamic analysis of the adsorption process were carried out, and the adsorption mechanism was characterized by SEM, FTIR and XRD. It is expected to provide a basis for the application and expansion of chitosan fiber in water treatment fields such as water purification or sewage treatment, and provide material support for the development of more water purification products.

## Materials and instruments

Materials: The CS fiber was purchased from Tianjin Zhongsheng Co., LTD. (china). The average length of CS fiber is 40 mm, and the linear density is 1.3D.

Copper sulfate pentahydrate, potassium chromate, ammonia water, ethyl alcohol (Aladdin Reagent Co., Ltd.), Escherichia coli, Staphylococcus aureus(Nanjing Lezhen Biotechnology Co.,Ltd.), Filter tube(Kunshan Huangda Plastic Products Co., Ltd.).

Instruments: UV spectrophotometer (model is SPECORD®210PLUS), The emission scanning electron microscope (SEM, TM330), X-Ray Diffractometer(XRD, D8 Discover).

## Experiments methods and fitted models

### Experimental test methods

Performance test of the CS fiber to remove Cr(VI) and Cu(II) ions was conducted within a flask with 250 mL capacity. The employed Cr (VI) and Cu(II) ions solution quantity was 100 mL in each experiment. The 0.1 mol/L HCL and NaOH were utilized for adjusting solution pH value. The speed and temperature of shaker kept at 90 rpm and 288 K, respectively. After reaction, the filtrate is collected for analyzing Cr(VI) and Cu(II) ions concentration. The experimental methods of Cr(VI) are presented in Table 1.

**Table 1:** Experimental methods in this study.

Set	Experiment factors	pH value	CS content(g)	Reaction time (min)	Original content (mg/L)
1	Effect of pH value	1/3/5/7 9/11/13	0.05	90	100
2	Effect of CS dosage	7	0.025/0.050/0.075/ 0.1/0.125/0.15	90	100
3	Effect of reaction time	7	0.05	5–180	100
4	Effect of initial concentration	7	0.05	90	50/75/100 125/150
5	Adsorption kinetic	7	0.05	5–180	50/100/150
6	Adsorption isotherm	7	0.05	90	50/75/100 125/150

### Determination of Cr(VI) and Cu(II) ions concentration

The adsorption capacity and adsorption efficiency of the fiber to Cr(VI) ions and Cu(II) ions are calculated by formulas (1), (2) and (3) respectively^22–24^.

1 $$q_{t} = \frac{{V(C_{0} - C_{t} )}}{1000 \times M}$$

2 $$q_{e} = \frac{{V(C_{0} - C_{e} )}}{1000 \times M}$$

3 $$E_{t} = \frac{{(C_{0} - C_{t} )}}{{C_{0} }} \times 100\%$$

In the formula: q_e_ represents the equilibrium adsorption capacity of Cu(II) and Cr(VI) (mg·g^−1^); q_t_ represents Adsorption amount of Cu(II) and Cr(VI) at time t (mg·g^−1^); E_t_ represents adsorption efficiency at time t (%); V represents Solution volume (mL); C_o_ represents the mass concentration of Cu(II) and Cr(VI) in the solution before adsorption (mg·L^−1^); C_e_ represents the mass concentration of Cu(II) and Cr(VI) in the solution at the time of adsorption equilibrium (mg·L^−1^); C_t_ represents the mass concentration of Cu(II) and Cr(VI) in the solution at time t (mg·L^−1^); M represents dry weight of CS fiber (g).

### Dynamic calculation

The quasi-first order kinetic equation is as follows:

4 $$Ln(q_{e} - q_{t} ) = Lnq_{e} - K_{1t}$$

5 $$\frac{t}{{Q_{t} }} = \frac{1}{{k_{2} q_{{\text{e}}}^{2} }} + \frac{1}{{{\text{q}}_{{\text{e}}} }}{\text{t}}$$

In the formula: q_e_ is the amount of Cu(II) or Cr(VI) adsorbed in the adsorption equilibrium; q_t_ is the amount of copper ion adsorption at time t; K_1_ is the adsorption rate constant of the quasi-first-order model (min^−1^); K_2_ is the adsorption rate constant of the quasi-two-stage model (g·mg^−1^·min^−1^).

## Results and discussion

### Effect of single factor on Cr(VI) and Cu(II) ions removal

**Figure 1:**
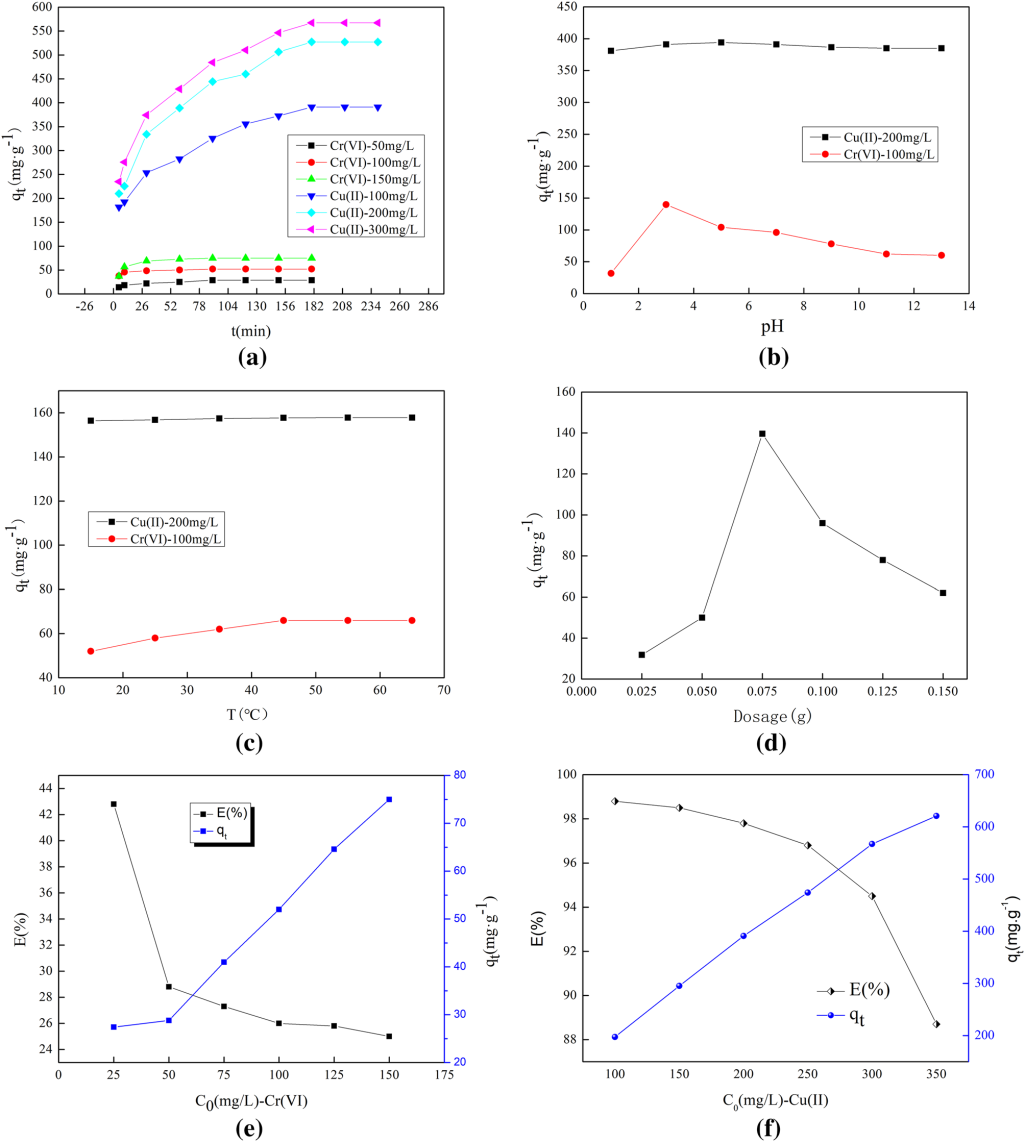
Varying factor effects: (**a**) Reaction time; (**b**) pH; (**c**) Temperature; (**d**) dosage as well as (**e**) (**f**) the initial concentration.

Figure 1a shows that the diffusion amount of copper ions by the shell fiber increases with time. The penetration of cellulose to ultraviolet ions is slow. The shell cellulose permeates the ion time, the first 60 min, the speed is faster, after 60 min, the absorption rate increases to 180 min, and the penetration equilibrium is reached. In the case of copper ions, the penetration rate is fast in the first 90 min, and after 90 min, the penetration rate reaches 180 min, which gradually tends to break the balance. When the concentration is 150 mg/L, the removal rate of Cr(VI) ions by CS fiber is 25.7%. When the final Cu(II) ion concentration is 200 mg/L, the removal rate of Cu(II) ions by CS fiber is 98.7%. The ion penetration of CS has a much higher penetration of Cu(II) than Cr(VI). This is mainly because the conductive group of CS fiber has permeability to divalent metals.

Figure 1b displays that as the pH value of the Cr(VI) ions solution increases, the adsorption capacity of the CS fiber to Cr(VI) ions increases first, and the adsorption capacity of the CS fiber is the largest when the pH is 3, so the optimal pH value is 3. When the pH of the solution is higher than 7, the absorption of Cr(VI) ions by the fiber begins to decrease. The existence form of Cr ions is affected, and it exists in the form of H2CrO4 or H2CrO4^−^ under obvious pH value. In the presence of neutral and alkaline conditions, in the form of CrO4^−^. It exists in the form of Cu^2+^ under acidic conditions and mainly in the form of Cu(OH)_2_ under alkaline conditions. In Fig. 1b, the best pH value for CS fiber to penetrate Cr(VI) ions is 3, and the best pH value for CS fiber to penetrate Cu(II) ions is 5. This is mainly because the pH value and the amount of H^+^ in the metal solution are different, resulting in different adsorption sites, and the two metal ions have different states under different pH environments. The existing form is Cr(VII), and the form of copper ions under corrosive conditions is Cu(II), so when the fiber oozes different metal ions, it has better permeability to copper ions. Compared with copper ions, battery ions are affected by the pH value because the battery has ions at a conditional price, and the particles in the shell fiber have better permeability to low-level metal ions. Therefore, when the fiber penetrates multiple metal ions at the same time, the penetration properties are diverse^25^.

Figure 1c indicates that as the temperature rises, the fiber's penetration of Cr(VI) ions slowly increases, and the penetration does not change significantly after 35 °C. 35 °C can be determined as the optimal temperature to heat the antenna fiber penetration. This phenomenon shows that when the casing increases the penetration of cellulose into battery ions, the temperature has cathodoluminescence ion penetration, that is, high fracture at high temperatures. The absorption process is an endothermic process. When the temperature lasts for a time (T > 55 °C), due to molecular movement and expansion of macromolecules, the micropores of the outer cellulose fibers decrease, which increases the retardation of the diffusion of chromium ions into the micropores. Suppresses the progress of cellulose swelling. Fiber penetration of copper ions is not significantly affected by temperature^26^.

Figure 1d shows the experimental effect of the amount of chitosan fiber on the removal of Cr(VI) ions. The figure shows that the removal rate increases as the amount of CS fiber increases. This is mainly because as the amount of CS fiber increases, the number of effective active sites that can react with Cr(VI) ions increases, so that there is more mutual reaction between chitosan fiber and Cr(VI) ions. The experiment on the effect of Cr(VI) ions concentration on Cr(VI) ions removal efficiency was explored. The results are shown in Fig. 1e. It can be clearly seen that the Cr(VI) ions removal efficiency decreases with the increase of Cr (VI) ions concentration. After the action time of chitosan fiber and Cr(VI) ions is 180 min, when the initial concentration of Cr(VI) ions is 25 mg/L, the best removal rate is 42.8%. The experimental results can show that CS fiber is more suitable for reaction with low concentration of Cr(VI) ions.

Figure 1e,f displays that the adsorption trend of the CS fiber to Cr(VI) ions and Cu(II) ions is the same, and as the metal concentration increases, the adsorption efficiency decreases. This is mainly because at lower concentrations, the ratio of the initial number of moles of metal ions to the effective surface area is lower, and subsequently, the adsorption fraction has nothing to do with the initial concentration. At higher concentrations, compared with the number of moles of metal ions present, the available adsorption sites of the adsorbent become fewer.

### Kinetic experiments

**Figure 2:**
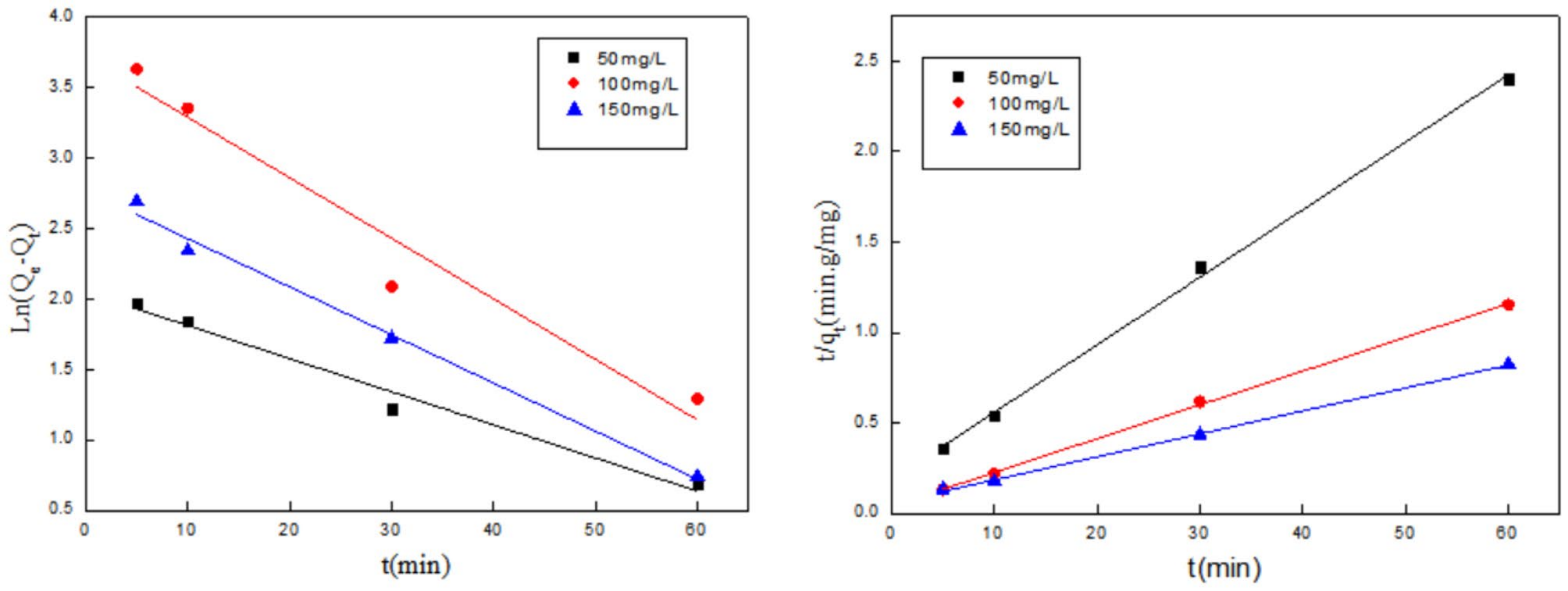
Quasi-first-order and quasi-second-order kinetic equations on adsorption of Cr(VI) ions.

As showed in Fig. 2 and Table 2. According to the value of R^2^, the value of R_2_^2^ is greater than 0.99, and the value of R_1_^2^ is less than 0.99, R_2_^2^ > R_1_^2^, it can be concluded that the adsorption of Cr(VI) ions is more in line with the second-order kinetics. Hence, pseudo-second-order kinetic equations are suitable for explaining adsorption profiles. The kinetic parameter R^2^ is less than 1, which indicates the favor adsorption profile.

**Table 2:** Kinetic model parameters of Cr(VI).

C(mg/L)	Quasi-first order kinetic equation		Quasi-second-order kinetic equation	
Set	Linear regression equation	R_1_^2^	Linear regression equation	R_2_^2^
50	y = − 0.0235*X + 2.0467	0.9713	y = 0.0373*X + 0.1869	0.9974
100	y = − 0.0429*X + 3.7166	0.9344	y = 0.0187*X + 0.0409	0.9991
150	y = − 0.0341*X + 2.7682	0.9833	y = 0.0127*X + 0.0593	0.9988

**Figure 3:**
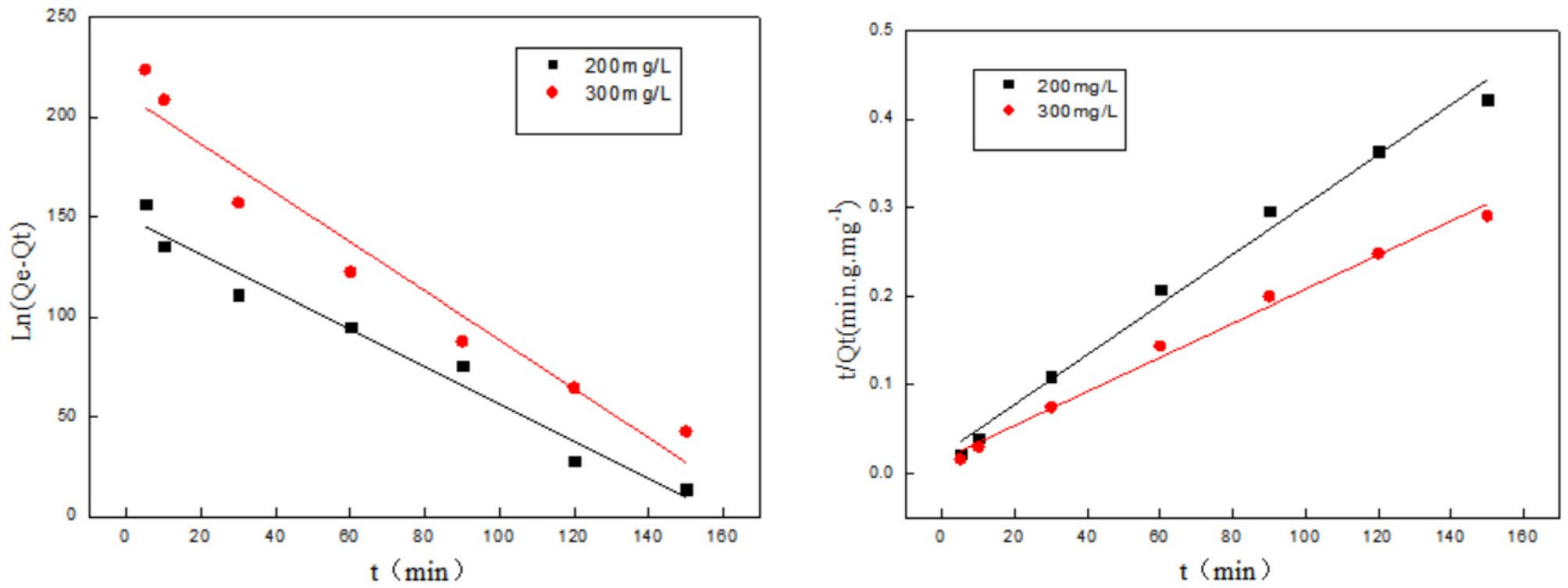
Quasi-first-order and quasi-second-order kinetic equations on adsorption of Cu(II) ions.

It can see from Fig. 3 and Table 3, the kinetic equation of the CS fiber for the adsorption of Cu(II) ions has the same properties as that of Cr(VI) ions. The kinetic simulation using pseudo-first-order is not good and the actual data deviates seriously from the fitted curve. At the same time, the correlation coefficient of kinetic parameters is low. While the pseudo-second-order kinetic plot, with high correlation coefficient of kinetic parameters.

**Table 3:** Kinetic model parameters of Cu(II).

C(mg/L)	Quasi-first order kinetic equation		Quasi-second-order kinetic equation	
	Linear regression equation	R_1_^2^	Linear regression equation	R_2_^2^
200	y = − 0.09322*X + 149.84	0.9662	y = 0.0028*X + 0.0217	0.9899
300	y = − 1.2226*X + 210.7589	0.9434	y = 0.0019*X + 0.0158	0.9877

### Isotherm experiments

**Figure 4:**
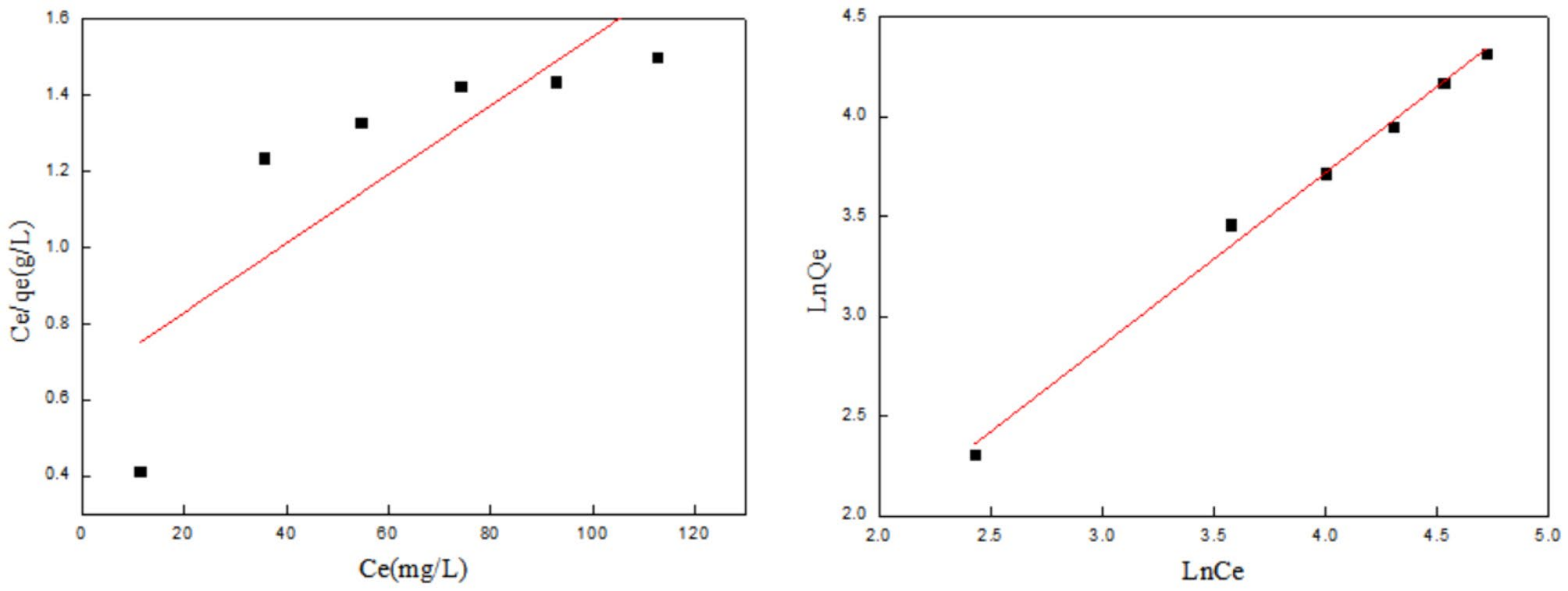
Adsorption isotherm of Cr(VI) ions.

From the parameters R_1_^2^ < R_2_^2^ in Fig. 4 and Table 4, we can see that the adsorption of Cr(VI) ions by chitosan fibers is more in line with the Langmuir model, and the adsorption process is multi-layer adsorption. At a temperature of 288 K, the maximum theoretical saturated adsorption capacity of chitosan fiber for Cr(VI) ions is 111 mg/g. Since 0 < R_L_ < 1, the adsorption process is favorable. According to Freundlieh model, parameter 1/n value is between 0 and 1, indicating that the experimental concentration range is beneficial for Cr(VI) adsorption onto CS fiber.

**Table 4:** Fitting parameters of adsorption isotherms of Cr(VI).

Langmuir						Freundlich		
Q_e_/mg/g	Q_m_/mg/g	K_L_	R_L_	R_1_^2^	K_f_		1/n	R_2_^2^
75	111	0.014	0.742	0.6062	195		0.863	0.9923

**Figure 5:**
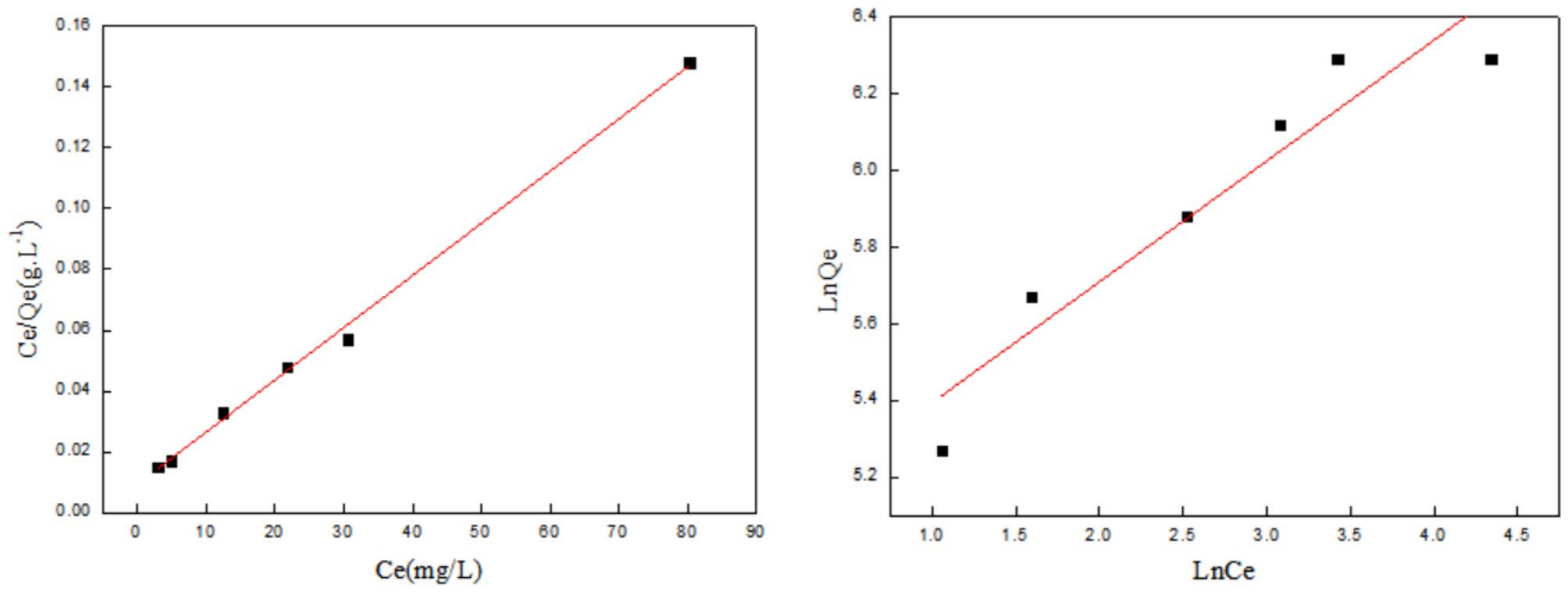
Adsorption isotherm of Cu(II) ions.

In addition, based on the analysis of Cr(VI) ions, we can get the Freundlich model to illustrate the non-ideal adsorption of non-uniform surface and multilayer adsorption. It can be seen from Fig. 5 and Table 5 that the high k_f_ value indicates the high affinity of the CS fiber to Cu(II) ions. This is also because the amino groups of chitosan fibers have better chelating properties with copper ions, and copper ions can be more stably adsorbed on the fibers.

**Table 5:** Fitting parameters of adsorption isotherms of Cu(II).

Langmuir						Freundlich		
Q_e_/mg.g^−1^	Q_m_/mg.g^−1^	K_L_	R_L_	R_1_^2^	K_f_		1/n	R_2_^2^
539.6	588.24	0.196	0.048	0.9966	195		0.315	0.8815

### Adsorption mechanism

#### SEM analysis

**Figure 6:**
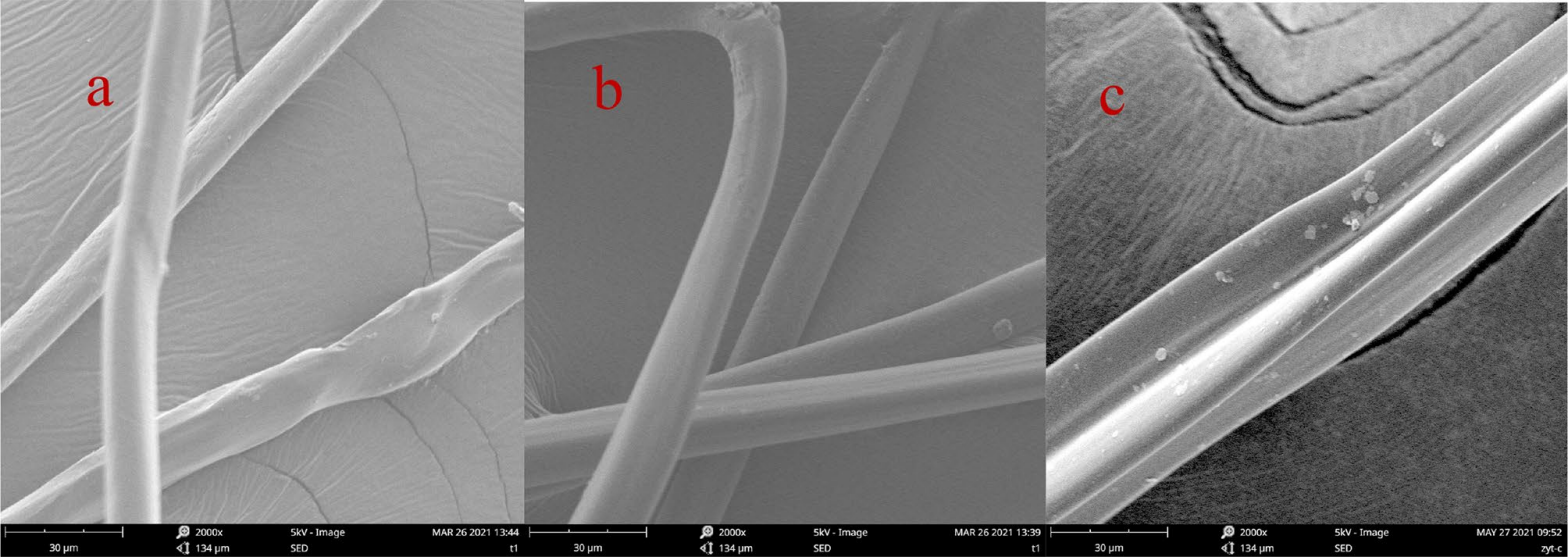
(**a**) CS SEM images before and (**b**) after the reactionwith Cr(VI) ionsand (**c**) with Cu(II) ions.

Figure 6a shows the surface of the CS material before adsorption is relatively smooth, Compare the surface of the material after adsorption of chromium(Fig. 6b) and copper(Fig. 6c), and the surface of the CS material after adsorption is rough, which is conducive to the adsorption of copper and chromium ions in the next step, Compare the surface of the material after adsorption of copper and chromium, the surface of the CS material after adorption with Cu(II) ions is more rough. A large amount of copper ions are uniformly distributed on the adsorbed material, which shows that the CS material has high adsorption for Cu(II) ions. In the study by H LI^26,27^ on the absorption of copper ions by grafted PTFE fiber, it was also proved that after the fiber adsorbed copper ions, the surface became rough. The main reason for this may be that the exudation of the shell cellulose and metal ions is on the fiber, Not is in the water phase, thus forming the shell cellulose to coagulate the metal ions and the surface is rough.

#### XRD results

**Figure 7:**
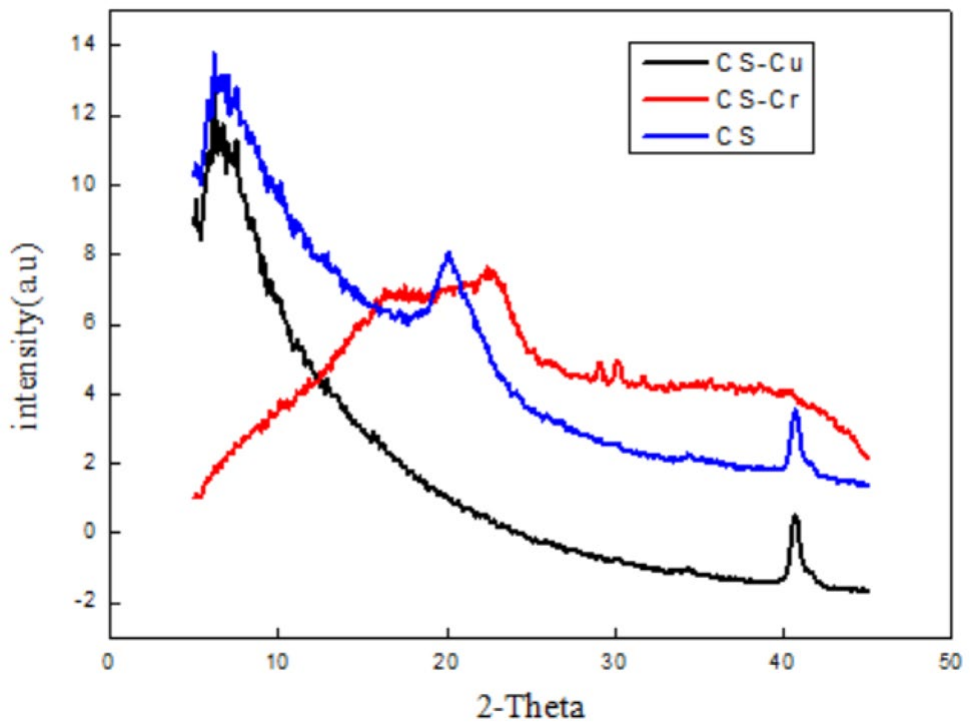
XRD of CS before and after use for Cr (VI) and Cu(II) ions adsorption.

AS show in Fig. 7. CS fiber has two different crystal forms, both of which belong to the monoclinic crystal system, namely Form I (2θ in10°) and Form II (2θ is about 20°). As can be seen from Fig. 5, CS fiber has a broad crystallization peak at 2θ = 10.9°, which represents the hydrated crystals of chitosan. It is due to the fact that water molecules enter the chitosan. After reacting with chromium, the peak at about 10° disappears, It shows that after the adsorption of CS molecules with chromium, the separation is weakened.The regularity of the three-dimensional structure of the sub-chain reduces the intramolecular crystalline area.

In addition, After contact with copper ions, the peak at about 20° disappears.This also indicates that the functional groups of chitosan fibers have chelated and cross-linked with copper ions. At the same time, after the transparent cellulose of the shell penetrates Cr(VI) ions and Cu(II) ions, the fixed volume of the adsorbed Cu(II) ions increases significantly, while the amorphous volume without Cu(II) ions is relatively, which shows that the shell is very cool. After sound and may interact with Cu(II) ions, it has the integrity of the molecular space three-dimensional network structure, which reduces the intramolecular crystalline area.

#### FTIR pattern

**Figure 8:**
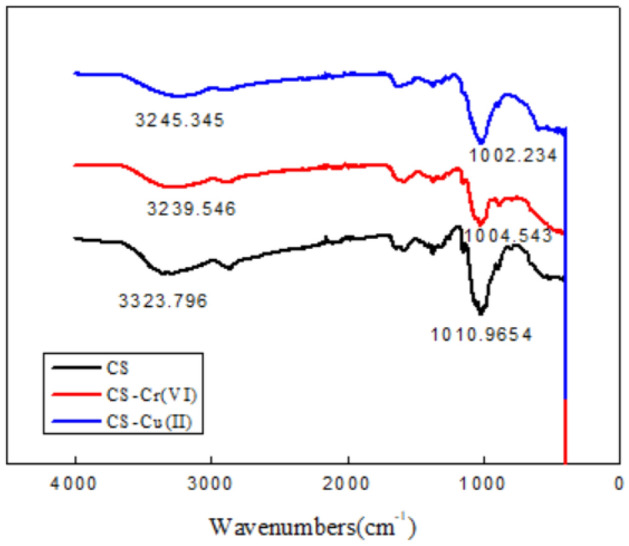
FTIR of CS before and after use for Cr (VI) and Cu(II) ions adsorption.

It can be seen from Fig. 8 that the infrared spectra of CS fibers before and after the adsorption of Cu(II) ions and Cr(VI) ions are roughly similar, and the position of the characteristic absorption peak is basically unchanged. After the formation of coordination bonds between O–H and Cu(II) ions in the fiber, the hydrogen bond between O–H is destroyed, and the addition of Cu(II) ions steric hindrance caused by Cu(II) ions indicates that Cu(II) ions has been complexed with the CS fiber. The broad absorption peaks between 3323.796 cm^−1^ to 3239.546 cm^−1^ and 3245.345 cm^−1^ in Fig. 8 are strong peaks produced by the stretching vibration of –OH and –NH. The peak area is obviously wider, and about 3300 cm^−1^ is shell aggregation. The O–H stretch vibration peak in the sugar fiber. Compared with the copper ion fiber, the chitosan fiber moves from 3280 cm^−1^ to 3175 cm^−1^ in the low frequency direction, and the intensity is weakened, indicating that Cu(II) and the hydroxyl O–H produce new, The hydrogen bonding effect destroys the original hydrogen bonding association^28,29^.

## Conclusion

This article provides a reference and basis for the preparation of new filter materials and products. The results are as follows:

Compared with Cr(VI) ions, Cu(II) ions can be better adsorbed by chitosan fibers. The removal rate of copper ions by chitosan fibers is 98.7%, and the removal rate of chromium ions is 42.8%. In the single-factor experiment of the adsorption of copper ions by chitosan fiber, the adsorption equilibrium time is 180 min, the optimal pH for adsorption is 5, and the optimal temperature is 35 °C. As the initial concentration of copper ions increases, the adsorption efficiency decreases. The adsorption of Cu(II) ions and Cr(VI) ions by chitosan fibers shows the same adsorption tendency.

Chitosan fiber adsorbs Cu(II) ions, and the adsorption process conforms to the second-order kinetic equation, and electrochemical adsorption plays a leading role. The adsorption isotherm is more in line with the Langmuir model. The adsorption process is multi-layer adsorption, which is a self-heating reaction that combines physical adsorption and chemical adsorption. The adsorption type is effective adsorption.

According to the SEM test results, it can be concluded that the surface of chitosan fiber adsorbs copper ions and becomes rougher. According to the results of FTIR and XRD, it can be concluded that the adsorption of heavy metal ions by chitosan fibers is mainly physical adsorption and chemical adsorption, and the functional group amino group is the adsorption functional group.

Chitosan fiber is soluble in acetic acid, and the spinning solution of chitosan fiber is under acidic conditions, which is beneficial to recovery. Spinning into chitosan fiber or other textiles, used in water treatment fields such as water purification, can remove harmful metal ions. It is an environmentally friendly material that can be continuously recycled and has broad application and development prospects.
